# Modified Nonnutrient Agar Plate Culture for the Diagnosis of *Strongyloides stercoralis* and Hookworm Infections in La-Ngu District, Satun Province, Southern Thailand

**DOI:** 10.1155/2022/1117400

**Published:** 2022-03-25

**Authors:** Sirima Kitvatanachai, Kanyanan Kritsiriwutthinan, Aree Taylor, Pochong Rhongbutsri

**Affiliations:** ^1^Faculty of Medical Technology, Rangsit University, Pathumthani 12000, Thailand; ^2^Department of Preclinical Science, Faculty of Medicine, Thammasat University, Pathumthani 12120, Thailand

## Abstract

Due to the low prevalence and light intensity of *Strongyloides stercoralis* (*S. stercoralis*, Ss) and hookworm (HW) in Thailand, an increase in the efficacy of detection with the appropriate diagnosis is needed. This cross-sectional survey is aimed at using modified nonnutrient agar plate culture (mNNAPC) to assess the prevalence of *S. stercoralis* and hookworm infections and to report intestinal parasitic infections in La-Ngu villagers, Satun Province in Southern Thailand. We used wet smear, modified Harada-Mori filter paper culture (mHMFPC), and mNNAPC to investigate 204 villagers (4 villages) between August 2016 and January 2017. The combination of mHMFPC and mNNAPC raised the prevalence of *S. stercoralis* and hookworm infections among the study population from 3.4% and 1.0% to 6.9% and 2.5%, respectively. There were no significant differences between demographic characteristics and these infections (*p* < 0.05). Three types of protozoal infections, *Blastocystis* spp. (2.9%), *Entamoeba histolytica*-like (0.5%), and *Giardia duodenalis* (0.5%), and 3 species of helminthic infections, *S. stercoralis* (6.9%) (14 cases), hookworm 2.5%, and *Enterobius vermicularis* 0.5%, were demonstrated in this area. The mNNAPC showed the highest efficacy in detecting both parasites (Ss 92.9% and HW 80%), whereas the wet smear detected none.

## 1. Introduction

*Strongyloides stercoralis* and hookworm are soil-transmitted helminths (STHs) that are widely distributed in tropical and subtropical areas, including Thailand [1, 2]. In immunocompromised patients, the parasite can cause autoinfection, which may lead to hyperinfection, with large numbers of larvae eventually causing disseminated strongyloidiasis [3]. Hookworm is a blood-feeding intestinal parasite, resulting in blood loss from the damaged gut wall. The effects of hookworm infection include problems in the development of children and increased mortality in pregnant women and their infants [4–6].

Several coprological methods have been used to detect larvae in stool samples, including direct fecal smear [7–9], the Baermann funnel [8], sedimentation methods [7, 10], filter paper culture [9], and agar plate culture [11–13].

Reported prevalence varies according to host status, study area, and diagnostic techniques used [14]. The traditional direct wet mount of stool has a low sensitivity in detecting light helminthic infections. In areas that use this wet mount technique, it may significantly increase misdiagnosis of intestinal parasites [15]. Wet mount is still first priority for screening parasitic infection in routine labs in hospitals of Thailand. Cultivation techniques such as Harada-Mori and agar plate culture have been used for large-scale surveys [16, 17]. In 11 provinces of Southern Thailand, 2007, using agar plate culture technique revealed that the overall prevalence of *S. stercoralis* was 20.6% and the intensity was mostly low, reported at 27.1% in some areas of Satun Province, and was never studied in La-Ngu District [18]. From 159 villagers in Krabi Province, the highest detection rate of hookworm and *S. stercoralis* was 11.3% and 3.1%, respectively, using modified Harada-Mori filter paper culture (mHMFPC), which was higher than direct wet mount (6.9% and 0%, respectively) [19]. mHMFPC detected more positive cases of hookworm than wet mount: 5 vs. 2 in Yo Island, Southern Thailand [9]. However, mHMFPC is still of limited use in field surveys when stool form is loose or diarrhea. The agar plate (APC) appears to be the more promising without this limitation.

Several modified APC (mAPC) have improved its sensitivity, using cellophane paper to preserve larval stages of parasites [11]. A mAPC, which is optimized for pH, temperature, salinity, and nutrient, could develop *S. stercoralis* larvae more than APC [20]. Recently, an optimized agar plate culture was used in an endemic community at Khon Kaen Province, Thailand, and the result revealed that the mAPC (11.99%) showed higher prevalence than APC (8.46%) [13]. Even though APC is cumbersome and time-consuming, this technique is still useful in areas with low intensity of these two parasites or in chronic infections, which will increase the true prevalence. In this study, we considered modified nonnutrient agar plate culture (mNNAPC) to increase probability for finding parasites in the fecal sample because it is easy to prepare nonnutrient agar, which is available at the local market, reducing costs of nutrient agar, and it is easier to process in a general laboratory. This might be a feasible and implementable routine wet mount for diagnosing *S. stercoralis* and hookworm in field survey or local hospitals. We studied an area of unknown prevalence, La-Ngu District, and reported the data regarding intestinal parasites, combining the results with wet mount and mHMFPC.

## 2. Materials and Methods

### 2.1. Study Area, Study Design, and Subject

**Figure 1:**
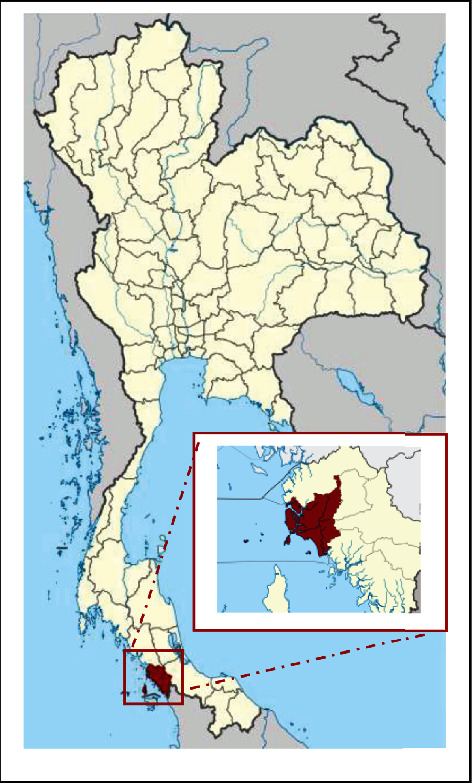
Map of La-Ngu District, Satun Province, Southern, Thailand (modified map from Wikimedia Commons: https://en.wikipedia.org/wiki/Satun_Province).

La-Ngu District was one of the seven districts of Satun Province, lower Southern Thailand (Figure 1), approximately 50 km from Muang Satun (6°51′50^″^N latitude and 99°48′9^″^E longitude). The district is divided into six subdistricts. There are two seasons, summer, which runs from February-May, and the rainy season. The majority of the population make a living from agriculture, fishing, and selling [21].

This cross-sectional study was conducted in La-Ngu District, Satun Province, from August 2016 to January 2017. This was approved by the Human Ethical Review Committee of Rangsit University, Thailand (ethical clearance No. RSEC 42/2558). The sample size was determined using the single population proportion formula. The prevalence rate (*p*) is of 16% from previous study [22], with a 95% confidence interval (*z* = 1.96) and 5% margin of error (*d* = 0.05). The sample size was finally calculated to be 206.

Villagers from 4 subdistricts (La-Ngu, Paknam, Bannaimuang, and Namphud) in all age groups and including males and females, were requested to provide stool samples collected by health workers in each village. People who did not provide sufficient stool specimens were excluded from the study. A labeled clean plastic container and the instruction how to collect the stool samples were distributed to participants. Sociodemographic data (gender, age, religion, education, occupation, and income) of participants were collected using a structured questionnaire. A standardized questionnaire was developed based on previous studies [9, 10], and to determine the content validity, index of item objective congruence (IOC) was investigated by 3 parasitologists. Pretested questionnaires were used to interviewed people outside 4 subdistricts from this study in Satun Province and tested the reliability with Cronbach's alpha correlation to adjust the questions.

### 2.2. Laboratory Methods and Stool Examinations

A single stool specimen was collected from each participant. Fresh stool samples were directly processed and examined following 3 methods.

#### 2.2.1. Wet Mount Preparation

Stool was immediately prepared in accordance with standard protocols [23]. One to two milligrams of stool was smeared on a slide with 0.85% NSS and 1% iodine. The wet mounts were examined under light microscope at 10x and 40x magnifications.

#### 2.2.2. The Modified Harada-Mori Filter Paper Culture (mHMFPC) (modified from Harada and Mori [9, 19, 24])

Briefly, 2 g of fresh stool was placed on a folded strip of filter paper (30 mm × 150 mm), which was then placed in 30 mm × 200 mm plastic tube containing 5 mL of sterile distilled water and incubated at room temperature (27–35°C) for 7 days. 0.5 mL formaldehyde was then added, and the tube was centrifuged at 2000 r/min for 5 min. The sediment was examined under the microscope. All third stage larvae were identified to species [25, 26].

#### 2.2.3. Modified Nonnutrient Agar Plate Culture (mNNAPC) (modified from Koga et al. [27])

Our pilot study in hookworm cases to compare mNNAPC with conventional APC (*n* = 30) showed similar positive agreement (100%) results in both techniques (unpublished data). Briefly, mNNAPC was perform by placing 2 g of feces on plastic dishes (diameter 8.5 cm and depth 1.5 cm) containing 5 mL of 1.2% nonnutrient agar (agar available in supermarket). Dishes were sealed with parafilm to prevent insects laying eggs or eating larvae and to prevent larvae from crawling out. Plates were incubated at room temperature (27–35°C) for 7 days in the dark box. The surface of agar was washed with 10% neutral buffer formalin and centrifuged at 2,500 rpm for 5 min to collect larval and adult forms of parasites [27]. The sediment was examined for hookworm and *Strongyloides* under the microscope. All third stage larvae or adults of hookworm and *Strongyloides* were identified to species [25, 26].

The result was considered as positive for parasitic infection when one of the techniques showed a positive result. All samples were blindly examined by two microscopists. Experienced parasitologists reread all positive samples and 10% of randomly selected negative samples.

### 2.3. Data Analysis

Data were processed using the IBM SPSS software for Windows (Version 21.0). Descriptive statistics were used to describe the prevalence of parasitic infections in each method. Chi-square test was used to compare the proportion of sociodemographic characteristic data and the hookworm and *S. stercoralis* infections. A *p* value < 0.05 was considered statistically significant.

The efficacy of the method was calculated as follows:

(1) $$\begin{array}{c} \text{Efficacy of the method}=\frac{\text{A number of positive case in each method}}{\text{A number of positive case in each method}}\times 100. \end{array}$$

## 3. Results

### 3.1. The Prevalence of Intestinal Parasitic Infections in La-Ngu District, Satun Province

A total of 204 participants from 4 villages La-Ngu District, Satun Province, participated in this study (Table 1). The total prevalence of intestinal parasitic infections using combined results of wet mount, mHMFPC, and mNNAPC was 12.3% (25/204) in La-Ngu District, Satun Province. There were 3 helminthic infections in this area; *S. stercoralis* showed the highest prevalence of 6.9% (14 cases), followed by hookworm 2.5% (5 cases) and *Enterobius vermicularis* 0.5% (1 case). Three species of protozoa were observed; *Blastocystis* spp. was the most dominant 3.4% (7 cases), followed by *Entamoeba histolytica*-like and *Giardia duodenalis* 0.5% (1 case) each. The highest prevalence of intestinal parasitic infections, 24%, showed in Paknam subdistrict (the range in the subdistrict being between 3.9 and 24.0% (Table 1)).

### 3.2. Sociodemographic Data of La-Ngu Villagers and Hookworm (HW) and S. stercoralis (Ss) Infections in La-Ngu District (*n* = 204)

There were a total of 204 participants, comprising 71 male and 133 female. The age-group distribution demonstrated that a majority (24.5%) were in the young group of 1-10 years, followed by age group 41-50 years (20.6%). About 80% of people living in La-Ngu District were Muslim, and more than half of the participants had completed secondary education. About thirty-seven percent of the participants worked as agriculturist and earned less than 9,000 baht a month (Table 2).

The sociodemographic characteristics of hookworm (HW) and *S. stercoralis* (Ss) infections are shown in Table 2. The difference in prevalence of hookworm (HW) and *S. stercoralis* (Ss) infections between genders, age groups, religions, education level, occupations, and incomes was not statistically significant (*p* > 0.05).

### 3.3. Hookworm and S. stercoralis Prevalence Rate by Diagnosis Techniques

Out of 204 participants who were subjected to investigate hookworm and *S. stercoralis,* wet mount method was unable to detect both hookworm and *S. stercoralis* (0.0%) in this area. Out of 5 positive hookworm cases, 3 and 4 cases were reported by using mHMFPC and mNNAPC techniques, respectively. *S. stercoralis* was reported in 8 and 13 cases (from 14 positive Ss) using mHMFPC and mNNAPC techniques, respectively. Overall, the prevalence of hookworm and *S. stercoralis* among patients using mHMFPC, mNNAPC techniques, and combined mHMC and mNNAPC techniques was 8.8%, 4.9%, and 7.8%, respectively (Table 3).

One case mixed infection between Hookworm and *S. stercoralis,* which was detected in both mHMFPC and mNNAPC.

### 3.4. The Efficacy of mNNAPC in Detecting Hookworm and S. stercoralis

The mNNAPC showed an efficacy of 80.0% (4/5) in detecting Hookworm, which was higher than mHMFPC 60.0% (3/5). The mNNAPC showed an efficacy of 92.9% (13/14), which was superior to the 57.1% (8/14) of mHMFPC in detecting *S. stercoralis.*

## 4. Discussion

Our study demonstrated a prevalence of intestinal parasitic infection of 12.3% in La-Ngu District, Satun Province, from 204 participants, using combined methods of wet smear, mHMFPC, and mNNAPC. There were 3 helminthic infections in this area, *S. stercoralis* (6.9%), hookworm (2.5%), and *E. vermicularis* (0.5%), and three species of protozoa, *Blastocystis* spp. (2.9%), *E. histolytica*-like, and *G. duodenalis* (0.5% each). The previous study, in Songkhla Province urban area, using wet mount combined with mHMFPC, demonstrated 0.8% of *S. stercoralis* and 2.4% of hookworm, which was a lower prevalence than this area [9]. No foodborne helminths were found in this area, which is similar to previous studies in Song Khla area [9]. This might be due to people living in Southern Thailand consuming cooked food. *Blastocystis* spp. was the most dominant (2.9%) of protozoal infections in this area. *Blastocystis* spp. is widely distributed in every part of Thailand. This study showed the prevalence of protozoa (2.9%) and *Blastocystis* spp. (2.9%) was higher than the previous report in 2008 [10], which might be because this protozoa can cause contamination through food and water; our interview reveals more than 50% of villagers drink water from natural sources such as pond, rain, and river without boiling it first.

The difference in prevalence of hookworm and *S. stercoralis* infections between sociodemographic characteristics, genders, age groups, religions, education level, occupations, and incomes, were not statistically significant (*p* > 0.05). This might be due to the low prevalence of infection.

The traditional routine wet mount was unable to detect both hookworm and *S. stercoralis* (0.0%) in this study. The low sensitivity of this method has been reported in many studies [8, 28, 29]. However, this method is still useful for intestinal parasite screening, especially protozoal infection such as *Blastocystis* spp. or *G. duodenalis* when using fresh stool specimen. It is also easy to prepare and costs almost nothing [9, 30]. Simple wet smear also reported a good result when used to diagnose patients with a high intensity of parasites in hospital [7]. In the area with a low intensity of parasite, we recommend this method should be considered with another potential method to increase the efficacy of detection. In this study, mNNAPC showed a superior prevalence rate to mHMFPC technique in detecting both hookworm and *S. stercoralis*, and combining mNNAPC and mHMFPC showed the highest prevalence of infections in La-Ngu villager, Satun Province. Agar plate culture is currently regarded as the most sensitive parasitological technique to diagnose *S. stercoralis* infection. This technique is also used to detect hookworm infection. However, this culture method has limitations due to the need for fresh stool. It is also cumbersome and time-consuming [8, 31–33]. Our study attempted to modify nonnutrient agar, which is available in local markets, to replace nutrient agar, in order to reduce costs of testing. In the future, the information gained from this study could be used to apply this practical method to field surveys for hookworm infection and *S. stercoralis* infection, to increase the efficacy of the detection in areas which have unknown prevalence.

## 5. Limitations

Due to the people in this district working in the early morning and not many people staying at home during our period of specimen collection, this study has limitations regarding using only a single stool specimen collection.

## 6. Conclusion

The wet mount was unable to detect both hookworm and *S. stercoralis* in this study, which might underdiagnose the prevalence. mNNAPC showed a higher efficacy in detecting both parasites, and nonnutrient agar testing can be easier to perform using locally sourced agar, resulting in low testing costs. Therefore, to increase the capacity of detection for hookworm and *S. stercoralis* or in the case of low intensity of these infections, we recommend mNNAPC be used in field survey.

## Figures and Tables

**Table 1:** The prevalence of intestinal parasitic infections in La-Ngu District, Satun Province.

Parasite	Study site				Total (%) (*n* = 204)
	La-Ngu (*n* = 52) No. (%)	Paknam (*n* = 50) No. (%)	Bannaimuang (*n* = 64) No. (%)	Namphud (*n* = 38) No. (%)	
Protozoa					
*Blastocystis* spp.	0 (0.0)	1 (0.5)	2 (1.0)	1 (0.5)	4 (2.0)
*Blastocystis* spp. and *Entamoeba histolytica*-like	0 (0.0)	0 (0.0)	1 (0.5)	0 (0.0)	1 (0.5)
*Giardia duodenalis* and *Blastocystis* spp.	0 (0.0)	1 (0.5)	0 (0.0)	0 (0.0)	1 (0.5)
Helminth					
*Enterobius vermicularis*	0 (0.0)	1 (0.5)	0 (0.0)	0 (0.0)	1 (0.5)
Hookworm	0 (0.0)	1 (0.5)	2 (1.0)	1 (0.5)	4 (2.0)
*Strongyloides stercoralis*	2 (1.0)	8 (4.0)	3 (1.5)	0 (0.0)	13 (6.5)
Hookworm and *Strongyloides stercoralis*	0 (0.0)	0 (0.0)	1(0.5)	0 (0.0)	1 (0.5)
Total	2 (3.9)	12 (24.0)	9 (14.1)	2 (5.3)	25 (12.3)

**Table 2:** Sociodemographic characteristics of hookworm (HW) and *Strongylodes stercoralis* (Ss) infections in La-Ngu District, Satun Province (*n* = 204).

Demographic characteristics	No. (%) (*n* = 204)	No. HW infections (%)	No. Ss infections (%)	Total HW and Ss infections (%)	*χ*2	*p* value
Gender					
Male	71 (34.8)	2 (2.8)	9 (12.7)	10 (14.1)	3.747	0.053
Female	133 (65.2)	3 (2.3)	5 (3.8)	8 (6.0)		
Age group (years)						
1-10	50 (24.5)	1 (2.0)	3 (6.0)	4 (8.0)	9.495	0.219
11-20	17 (8.3)	0 (0.0)	1 (5.9)	1 (5.9)		
21-30	8 (3.9)	0 (0.0)	0 (0.0)	0 (0.0)		
31-40	24 (11.8)	2 (8.3)	2 (8.3)	4 (16.7)		
41-50	42 (20.6)	0 (0.0)	2 (4.8)	2 (4.8)		
50-60	29 (14.2)	1 (3.5)	3 (10.3)	4 (13.8)		
>60	21 (10.3)	0 (0.0)	2 (9.5)	2 (9.5)		
No answer	13 (6.4)	1 (7.7)	1 (7.7)	1 (7.7)		
Religion						
Buddhism	15 (7.4)	0 (0.0)	0 (0.0)	0 (0.0)	2.893	0.235
Islam	163 (79.9)	4 (2.5)	14 (8.6)	17 (10.5)		
No answer	26 (12.7)	0 (0.0)	0 (0.0)	1 (3.9)		
Education						
No education	22 (10.8)	0 (0.0)	0 (0.0)	0 (0.0)	8.594	0.072
Primary and secondary						
School/high vocational/						
College/	116 (56.9)	4 (3.5)	13 (11.2)	17 (14.7)		
Bachelor degree and above	10 (4.9)	0 (0.0)	0 (0.0)	0 (0.0)		
No answer	56 (27.4)	1 (1.8)	1 (1.8)	1 (1.8)		
Occupation						
No work	38 (18.6)	1 (2.6)	1 (2.6)	1 (2.6)	2.408	0.661
Agriculturist	76 (37.3)	2 (2.6)	10 (13.2)	12 (15.8)		
Other	75 (36.8)	2 (2.7)	3 (4.0)	5 (6.7)		
No answer	15 (7.3)	0 (0.0)	0 (0.0)	0 (0.0)		
Income (baht)/month						
<9,000	76 (37.2)	3 (4.0)	9 (11.8)	12 (15.8)	9.255	0.055
9,001-16,000	14 (6.9)	0 (0.0)	2 (14.3)	2 (14.3)		
16,001-30,000	7 (3.4)	0 (0.0)	0 (0.0)	0 (0.0)		
>30,000	2 (1.0)	0 (0.0)	0 (0.0)	0 (0.0)		
No answer	105 (51.5)	2 (1.9)	3 (2.9)	4 (3.8)		
Total	204	5 (2.5)	14 (6.9)	18 (8.8)		

**Table 3:** Prevalence of hookworm and *S. stercoralis* infections in *La-Ngu villagers* using simple wet mount, mHMFPC, and mNNAPC.

Parasites	No. positive (%)			Total
	Wet mount	mHMFPC	mNNAPC	
Hookworm	0 (0.0)	3 (1.0)	4 (2.0)	5 (2.5)
*S. stercoralis*	0 (0.0)	8 (3.4)	13 (6.4)	14 (6.9)
Total	0 (0.0)	10 (4.9)	16 (7.8)	18 (8.8)

## Data Availability

The data used to support the findings of this study are included within the article.

## Abbreviations

HW: Hookworm
Ss: *Strongyloides stercoralis*
APC: Agar plate culture
mHMFPC: Modified Harada-Mori filter paper culture
mNNAPC: Modified nonnutrient agar plate culture.
